# The Association between Hair Cortisol and Self-Reported Symptoms of Depression in Pregnant Women

**DOI:** 10.1371/journal.pone.0161804

**Published:** 2016-09-01

**Authors:** Ellen Wikenius, Vibeke Moe, Marian Kjellevold, Lars Smith, Robert Lyle, Rune Waagbø, Christian Magnus Page, Anne Margrethe Myhre

**Affiliations:** 1 Institute of Clinical Medicine, Faculty of Medicine, University of Oslo, Oslo, Norway; 2 Department of Psychology, Faculty of social sciences, University of Oslo, Oslo, Norway; 3 The Center for Child and Adolescent Mental Health, Eastern and Southern Norway (RBUP), Oslo, Norway; 4 National Institute of Nutrition and Seafood Research (NIFES), Bergen, Norway; 5 The Faculty of Mathematics and Natural Sciences, School of Pharmacy, University of Oslo, Oslo, Norway; 6 Department of Medical Genetics, Oslo University Hospital, Oslo, Norway; 7 Child & Adolescent Mental Health Research Unit, Oslo University Hospital, Oslo, Norway; University of Oxford, UNITED KINGDOM

## Abstract

Depression has been linked to an imbalance in cortisol. Until recently, cortisol has been studied by measuring concentrations at single time points in blood or saliva samples. Cortisol concentrations vary with circadian rhythm and experiences, from time point to time point. The measurement of hair cortisol concentration (HCC) is a new method of accessing mean, long-term cortisol concentrations. Recent studies show positive associations between depression and HCC, and prenatal maternal cortisol is thought to influence the developing fetus. We therefore examined the association between HCC and self-reported symptoms of depression in second trimester pregnant women. Participants were 181 women, recruited between September 2011 and October 2013 to the Little-in-Norway (LiN)-study. These women answered the Edinburgh Postnatal Depression Rating Scale (EPDS) on self-reported symptoms of depression, and one cm maternal scalp hair was collected and analyzed for cortisol concentrations. Multiple regression analyses did not show depressive symptoms as a predictor for HCC in our selection of pregnant women, while gestational age was significantly related. In conclusion, our study indicated that symptoms of depression during pregnancy did not predict HCC, but further studies of clinically depressed, pregnant women using gestational age as an adjustment variable are warranted.

## Introduction

When the human body experiences stress, a cascade reaction starts originating in the hypothalamus, through the hypothalamic-pituitary-adrenal (HPA) axis, and resulting in the release of stress hormones, allowing the body to cope. Dysregulation of the HPA axis may be caused by severe and unpredictable stress [1]. The most studied stress hormone is cortisol. Until recently, cortisol has been studied by measuring concentrations at single time points in blood or saliva samples. A new method using hair samples for analyzing long-term cortisol concentrations has been developed [2]. Hair grows at different rates, depending on age, gender and individual differences; still, the general acceptance is that the mean human scalp hair growth is approximately one cm per month [3–5]. The extraction of cortisol from one cm hair can therefore be regarded as representative of one-month mean cortisol concentration retrospectively [6–10].

This new method of cortisol measurement was first used to show an increase in long-term hair cortisol concentrations (HCC) related to stress in macaque monkeys [11]. HCC has been studied as a possible biomarker of stress in humans [12, 13] and more specifically in psychiatric diseases [9]. The measurement of HCC may constitute an important, long term biomarker of stress [14]. Newer studies have established age and gender [15], season, hair characteristics, sociodemographic factors, health and body mass index [16, 17] as determinants of HCC to be included when studying HCC.

Depression can be regarded as a stress-related disorder and has been associated with an imbalance in cortisol levels [18, 19]. Associations between depression and HCC have been claimed in several recent studies [9, 20–22]. Dettenborn *et al*. [20] found increased HCC in 23 depressed, adult patients compared to 64 healthy adults, and Gerber *et al*. [21] found HCC predictive of depressive symptoms in 41 young adults. Wei *et al*. [23] compared 35 females with first- or recurrent-episodic depressions to 30 healthy female controls, and found significantly higher HCC in the females with first-episodic depression. An association between HCC and depressive symptoms was also observed in 20 caregivers of dementia patients compared to 20 healthy controls [22], while depressive symptoms were not found to predict HCC in two other studies [24, 25]. A systematic review by Staufenbiel *et al*. [9] on hair cortisol and chronic stress concluded that there was an association between major depression and HCC.

A review by Bennett *et al*. [26] on the prevalence of depression during pregnancy, found that pregnancy was associated with an increased risk of maternal depression. The risk increased from 7–9% in the general female population to 12.8% during the second trimester of pregnancy. Maternal cortisol increases naturally during pregnancy [27], and is therefore a biological link between mother and fetus by crossing the placenta [28]. If maternal cortisol increases in response to depression, it would pass through the placenta to the fetus [29].

Epidemiological studies have linked maternal stress during pregnancy to increased risk of poor physical health of the offspring later in life [30, 31]. Cortisol has been suggested as a biological link for this programming of the offspring [29]. Higher levels of maternal cortisol during pregnancy have been associated with lower IQ scores in children [32]. Timing of prenatal cortisol exposure has been found to be important for these effects; elevated cortisol early in pregnancy was associated with lower mental development scores, while exposure late was associated with higher scores [33]. Kalra *et al*. [12] found a positive correlation between perceived maternal stress and HCC in a study of 25 healthy, pregnant women. Investigating HCC as a biomarker of stress in pregnancy is therefore of interest for a wider understanding of the development of the fetus and later in life.

We hypothesized that a higher score of self-reported symptoms of depression in pregnant women was associated with higher HCC, with gestational age as an important adjustment variable. In the current study, hair samples and EPDS sum score data were collected on the same day. Hair samples represent the average cortisol levels for the past month while EPDS sum scores were based on the past seven days. Based on depressive diseases having long case histories [34], we assumed that the EPDS score represented symptoms of depression during the period of hair growth, and examined the association between self-reported symptoms of depression and HCC in 181 second trimester pregnant women.

## Materials and Methods

### Subjects

The participants of our study are part of a larger study population of pregnant women from the Little-in-Norway (LiN) study. LiN is a community-based population study with a prospective cohort design, investigating factors influencing child development [35]. The LiN study had midwives at nine well-baby clinics throughout Norway and asked all pregnant women between September 2011 and October 2013 to participate. Specially trained public healthcare nurses collected the data. The goal of the LiN study was to enroll approximately 1000 pregnant women who came for pregnancy check-ups at the nine sites during the enrollment period. The inclusion terminated when 1041 women had consented to participate. Five families later withdrew their consent, leaving 1036 prospective mothers.

The inclusion criteria for the present study were second-trimester, pregnant women participating in the LiN study, with saliva samples collected from the baby at 6 weeks for future analysis, leaving 364 participants. Further criteria for inclusion were singleton pregnancies (n = 361). We excluded one sample for having cortisol level higher than the possible cortisol detection level of 660 pg/ml, and all 18 hair samples from one of the nine sites, due to the use of hand cortisol cream by the research assistant at this site during sampling. Thus, 341 participants were included in this study (n = 341).

The Norwegian Regional Ethics Board has approved the study, and all participants gave written informed consent.

### Hair sample collection and measurements

Hair strands were cut near the scalp from the posterior vertex of the head after both blades of the scissors had been thoroughly cleaned with an alcohol swab. Dental floss was tied near the head end of the hair to mark the direction of hair growth and the hair samples were put into small plastic bags. The samples were stored in a freezer (-20°C) pending analysis.

Hair samples of 1–3 cm weighing between five and 13 mg were cut for analysis. The hair was analyzed for cortisol in two batches using the commercially available radioimmunoassay (RIA) kit, GammaCoat^™^ Cortisol ^125^I radio immune assay kit (DiaSorin CA1529E). Prior to analysis, the hair samples were homogenised and extracted in methanol [14]. The following modifications were used: hair samples were weighed (Mettler Toledo AX205 delta range), placed in Eppendorf tubes with a 5 mm steel ball. The tubes were frozen in liquid nitrogen for approximately 5 minutes before the hair was crushed in a Retsch MM 301 homogeniser (17 rpm), and repeated once, to produce fine hair powder. The tubes were centrifuged at 3000*g* for 1 minute and 1 ml methanol (Chromasolv, Sigma-Adrich) was added. The samples were then mixed on a Stuart rotator for 24 hours at room temperature, followed by centrifugation at 3000*g* for 10 minutes. Then, 700 μl supernatant was transferred to a new Eppendorf tube for evaporation of the methanol in a vacuum desiccator (30°C, 4–8 h). The samples were dissolved in 150 μl phosphate buffer and cortisol was analyzed using GammaCoat^™^ Cortisol ^125^I radio immune assay kit following the manufacturer’s instructions. The HCC, reported as pg/mg, was calculated from the analyzed cortisol concentration (nmol/L), dilutions, unit conversion, and weighed hair sample. The lowest and highest detectable concentration of cortisol in hair samples were 5 pg/mg and 660 pg/mg, respectively.

### Measurements of depression

Edinburgh Postnatal Depression Scale (EPDS) is a self-reported scale measuring psychological depression, which was answered by the pregnant women at the time of hair sample collection. EPDS is a commonly used screening tool for detecting depression in the postpartum period [36], and it has also been validated for use during pregnancy [37, 38]. Self-reported symptoms of depression during the previous seven days was registered, and the 10 items were scored from a score of zero, indicating no symptoms, to a score of three, indicating more severe symptoms. A total depression score (EPDS sum) is calculated, and the result ranges from zero to 30. Higher scores indicate more self-reported symptoms of depression [36].

The cut-off for the EPDS sum score for depression varies both between studies and countries [37]. The first EPDS study by Cox et al. recommended a cut-off of 10 for community surveys and screening, while a cut-off of 13 was seen as more appropriate for a clinical setting [36]. A cut-off of 11 was used by Berlé et al. when validating the Norwegian version of the EPDS [39], and therefore this is the cut-off used in this study.

### Socio-demographic variables

The participants volunteered information about age, education level and marital status. Data on fetal gender were collected from birth records.

### Statistical analyses

Descriptive statistics showed that there were no missing values in the variables. As cortisol measurements are often positively skewed [7, 40, 41] the cortisol data was log transformed (base 10). The log transformation provided close to normally distributed data for our sample. For descriptive purposes, mean values and standard deviations were presented in original HCC units (pg/mg). After inspection of the descriptive statistics, multiple regression analysis was performed with log HCC as the dependent variable, and EPDS sum as the main independent variables. The analysis was adjusted for gestational age, batch, fetal gender and maternal age. A p-value of <0.05 was considered statistically significant. The statistical analyses were done using SPSS version 22.

## Results

### Descriptive

The mean age of the pregnant participants was 30.2 years (SD = 4.9) Table 1, 54% were carrying boys and 46% girls. In total 80% had completed higher education; 1% had below upper secondary education (10 years or less), 19% upper secondary education (13 years), 38% higher education short (3 years at college or university level) and 42% higher education long (4 years or more at university level).

**Table 1:** Descriptive statistics of socio-demographic variables.

Socio-demographic characteristics	N (%) or Mean (SD) (Total N = 181)
Maternal age^a^ (SD)	30.2 (4.9)
Maternal educational level (%)	
Primary education	2 (1%)
Secondary education	34 (19%)
College	69 (38%)
University	76 (42%)
Maternal marital status (%)	
Married	61 (34%)
Living with partner	112 (62%)
Single	6 (3%)
Other	2 (1%)
Fetal gender (%)	
Girl	83 (46%)
Boy	98 (54%)
Batch	
Batch 1	174 (96%)
Batch 2	7 (4%)

^a^Range 20–43 years

### Hair cortisol concentration

Mean HCC for the 341 participating pregnant women was 63.0 pg/mg (SD = 27.0). The hair length of the hair samples varied; 181, 24, 99 and 37 samples being 1, 1.5, 2 and 3 cm long or longer, respectively. Hair length and cortisol levels were strongly correlated (p-value = 0.001). Thus, in further statistical analysis only samples of 1 cm were used (n = 181).

Mean HCC for the 181 participating pregnant women was 60.1 pg/mg (SD = 26.3). Of the hair samples, 174 were analyzed in the first HCC extracting batch in the laboratory and seven in a second batch.

### Depression scores and gestational age

The mean gestational age of the pregnancies at data collection was 24.8 weeks (SD = 3.9), the mean sum of self-reported symptoms of depression was 4.5 (SD = 3.8) Table 2. Of these 12 participants scored at or above the cut-off for depression.

**Table 2:** Descriptive statistics of the main study variables.

Study variables	N (181)	Mean (SD)	Range
Hair Cortisol Concentration (pg/mg)	181	60.1 (26.2)	25.9–280.6
Depression score (EPDS sum)	181	4.4 (3.7)	0–22
Gestational age (week)	181	24.8 (3.9)	17–32

### Main analyses

**Fig 1:**
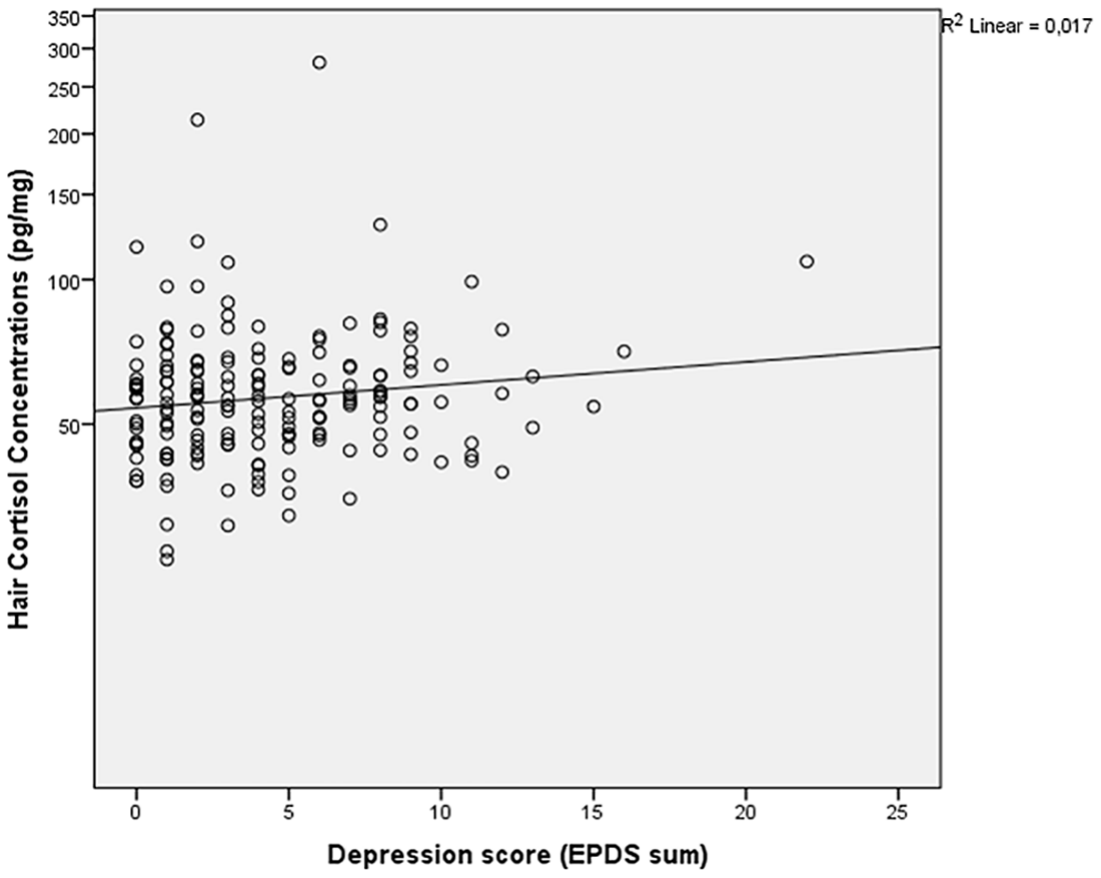
Hair cortisol concentrations (pg/mg) and depression scores (EPDS sum). The relationship between hair cortisol concentrations (pg/mg) and depression scores (EPDS sum) showed a non-significant Pearson correlation of 0.096.

**Fig 2:**
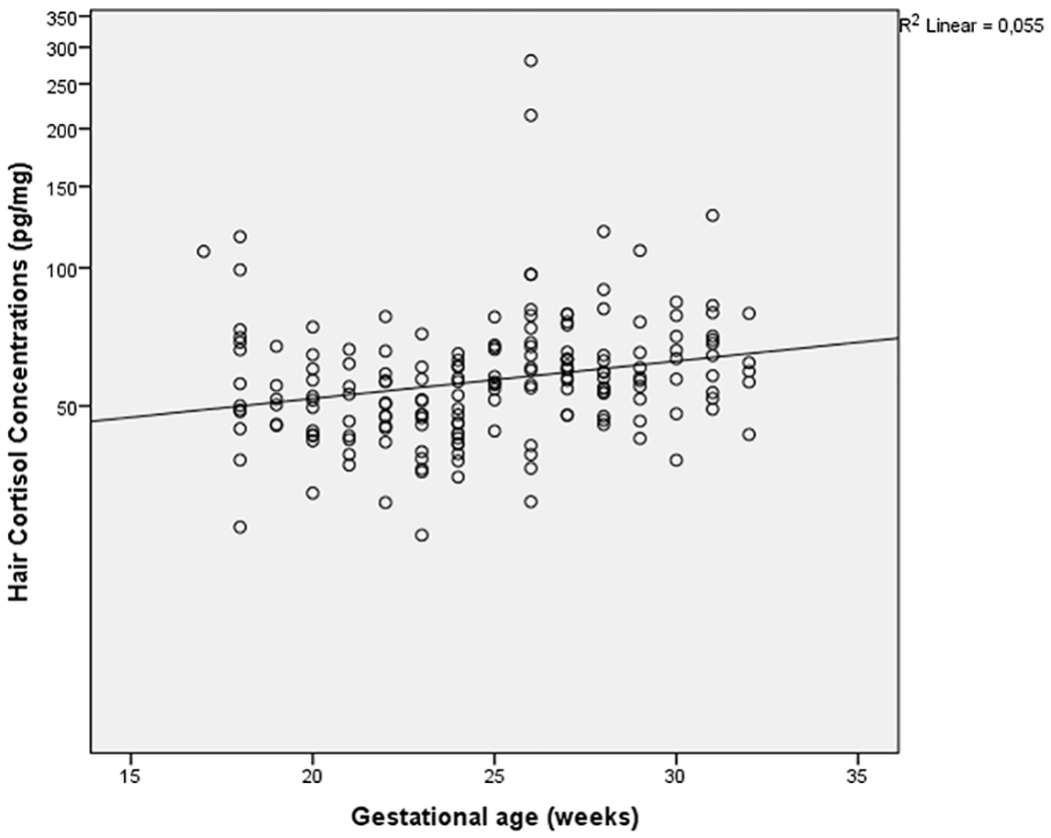
Hair cortisol concentrations (pg/mg) and gestational age (weeks). The relationship between hair cortisol concentrations (pg/mg) and gestational age (weeks) showed a Pearson correlation of 0.168 with a p-value of 0.024.

Scatter plots of HCC and depression scores illustrated a non-significant Pearson correlation of 0.096 (Fig 1). HCC and gestational age showed a Pearson correlation of 0.168 with a p-value of 0.024 (Fig 2).

Multiple regression analyses were performed with log HCC as the dependent variable, EPDS sum score as the main independent variables and gestational age, batch, fetal gender and maternal age as adjustment variables. The analyses were performed with maternal education, self-reported maternal health problems and season as adjustment variables without changing the findings. We did not find a statistically significant increase in HCC with depression score (p = 0.378). The analyses showed a significant difference in HCC by batch (p = 0.030) and a significant increase with gestational age (p = 0.002) Table 3. The results did not change when we excluded the seven samples from the second batch.

**Table 3:** Multiple regression analyses of log HCC by EPDS sum score, gestational age, health during pregnancy, fetal gender and maternal age (N = 175).

Variable (N = 181)	Type III Sum of Squares	F	Mean Square	p-value
Depression Score (EPDS sum)	1.592	1.081	0.100	0.378
Gestational age	0.938	10.186	0.938	0.002
Batch	0.439	4.768	0.439	0.030
Fetal gender ^a^	0.011	0.121	0.011	0.728
Maternal age ^b^	0.050	0.544	0.050	0.462

^a^Per 10 years

^b^Boy in relation to girls

## Discussion

In this study, we hypothesized that a higher score of self-reported depression symptoms in pregnant women was associated with higher HCC. Our findings did not support the growing evidence for associations between depression and HCC in recent studies [9, 20, 22, 23].

Our data did not show an association between self-reported symptoms of depression during pregnancy and HCC. Nor did it show an association between maternal age or fetal gender and HCC. However, we found that gestational age was significantly associated with HCC.

Dettenborn *et al*. [20] studied medicated in-patients with severe forms of depression compared with healthy controls, while we studied self-reported symptoms of depression in a general population of pregnant women. Our findings were inconsistent with their findings of associations between depression and HCC, raising the question whether severity of the disorder might affect associations between depression and HCC. A recent review by Vives *et at*. [42] found some support for associations between major depressive disorder and HCC, but in general, the findings in psychiatric disorders were inconsistent.

There is a natural increase in maternal cortisol during pregnancy, and this increased level is needed for fetal development [27, 33]. In our study, as found by Kirschbaum et al. [27], gestational age correlated with HCC. Kalra *et al*. [12] found a significant association between perceived stress and HCC in 25 healthy pregnant women, without adjusting for gestational age. Due to the natural increase in HCC during pregnancy, it would be of interest to study these findings with gestational age as an adjustment variable.

Many studies [20, 43, 44] have found reduced cortisol concentrations in hair in more distal hair segments, only one study did not find distal reduction in cortisol [20], and one found that hair cortisol concentrations increase naturally during pregnancy [27]. In the present study, cortisol concentration was significantly related to hair length. To reduce this uncertainty of variation in cortisol concentrations with hair lengths we chose to analyze one cm samples. Newer studies have shown health and seasons [15, 16] as determinants of HCC. We did not find correlations between seasons or maternal self-reported health problems or seasons and HCC in our sample.

Our study had certain limitations. Firstly, the participating women reported few depressive symptoms. Bennett *et al*. [26] found a mean prevalence of depression during the second trimester of pregnancy of 12.8%. With our 181 participants, one would expect approximately 23 women scoring at or above cut-off for depression. In our population, only 12 women scored at or above cut-off for depression, limiting our sample to mostly non-depressed pregnant women. Secondly, most of the women who participated in our study had completed higher education. In comparison, in the general Norwegian female adult population, only 32.5% have completed higher education [45], thus limiting the generalizability of the results.

In our study, maternal age and infant gender were not significantly associated with HCC, but gestational age was significantly associated with HCC. The finding of increase in HCC with gestational age is consistent with the current literature, while our null finding of HCC and depression is not. Our findings may specifically apply to a relatively healthy and well-educated population of individuals, or it may indicate that HCC is not associated with depression. In addition, this raises the question whether HCC is a long-term, biological marker of depression in pregnancy.

The present study did not support the hypothesized association between elevated levels of hair cortisol and increased symptoms of depression in pregnancy. However, one should consider the fact that just a few of the participants scored high on the depression rating scale. A sample consisting of more women with higher ratings of depression might yield different results. The results from our study should not be generalized to clinically depressed, pregnant women, but can be used to highlight the need for including gestational age in further studies of cortisol concentration as a biomarker of depression in pregnant women. In conclusion, depressive symptoms did not significantly predict HCC, while gestational age had a significant predictive value in our selection of pregnant women. Gestational age could therefore be an important adjustment variable when studying HCC during pregnancy.
